# The KRAB Domain‐Containing Protein ZFP961 Represses Adipose Thermogenesis and Energy Expenditure through Interaction with PPAR*α*

**DOI:** 10.1002/advs.202102949

**Published:** 2021-11-07

**Authors:** Lei Huang, Pengpeng Liu, Qiyuan Yang, Yong‐Xu Wang

**Affiliations:** ^1^ Department of Molecular, Cell and Cancer Biology Program in Molecular Medicine University of Massachusetts Medical School 364 Plantation Street Worcester MA 01605 USA

**Keywords:** diabetes, KRAB domain, obesity, PPAR*α*, repressor, thermogenesis, white fat browning, ZFP961

## Abstract

Adipose thermogenesis plays a pivotal role in whole‐body metabolic homeostasis. Although transcriptional mechanisms that promote thermogenesis are extensively studied, the negative regulatory network is still poorly understood. Here, a Krüppel‐associated box (KRAB) domain‐containing zinc finger protein, ZFP961, as a potent repressor of the thermogenic program is identified. ZFP961 expression is induced by cold and *β*3‐adrenergic agonist in adipose tissue. ZFP961 represses brown fat‐selective gene expression and mitochondrial respiration without any effect on general adipogenesis in cultured adipocytes. Adipose‐specific knockdown and overexpression of ZFP961 produce remarkable and opposite phenotypes of white fat remodeling. ZFP961 knockout mice display robust inguinal white adipose tissue browning, which is abolished by reexpression of full‐length ZFP961, but not by KRAB domain‐deleted ZFP961 mutant. ZFP961‐deficient mice are cold tolerant and resistant to high‐fat diet‐induced obesity, hyperglycemia, and hepatic steatosis. ZFP961 suppresses thermogenic gene expression by directly interacting with PPAR*α* and blocking its transcriptional activity, which can be completely negated by the PPAR*α* agonist. The findings uncover ZFP961 as a critical physiological brake that limits adipose thermogenesis and provides insights into the regulatory mechanisms that maintain energy balance and tissue homeostasis.

## Graphical abstract

This study provides new insights into the negative feedback mechanisms that sustain whole‐body energy balance and tissue homeostasis. Krüppel‐associated box domain‐containing zinc finger protein ZFP961 serves as a critical physiological brake to safeguard adipose thermogenesis. ZFP961 interacts with PPAR*α* to repress thermogenic gene expression and prevent energy over‐consumption.

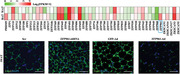

## 1 Introduction

Adipose tissue plays a crucial role in glucose homeostasis and energy balance. Whereas white adipose tissue (WAT) is responsible for energy storage in times of excessive nutrients, brown adipose tissue (BAT) is characterized by dissipating energy as heat in a process called non‐shivering thermogenesis,^[^ ^1^ ^]^ which is primarily executed by mitochondrial oxidative metabolism and uncoupling protein 1 (Ucp1)‐mediated uncoupling from ATP production,^[^ ^2^ ^]^ although Ucp1‐independent pathways have also been identified recently.^[^ ^3^ ^]^ Certain WAT depots are highly recruitable and can be induced to brown‐like (beige) adipocytes en masse under external stimuli.^[^ ^4^ ^]^ Given that both classical BAT and beige fat are present in adult humans and their activity is inversely associated with body mass index and age,^[^ ^5^, ^6^ ^]^ there is significant interest in developing strategies to boost adipose thermogenesis as potential therapeutic measures for obesity and associated metabolic diseases.^[^ ^7^, ^8^, ^9^, ^10^ ^]^ On the other hand, adipose thermogenesis must be tightly regulated to prevent unwanted or detrimental energy‐wasting; this is particularly important in conditions such as limited food availability, cancer cachexia, burn injury, hyperthyroidism, and infection.^[^ ^11^, ^12^, ^13^, ^14^, ^15^, ^16^ ^]^

Pathways and mechanisms that promote thermogenesis have been comprehensively illuminated.^[^ ^17^ ^]^ These studies have revealed that control of thermogenesis principally occurs at the transcriptional level,^[^ ^17^, ^18^, ^19^ ^]^ and a number of critical transcriptional regulators have been identified, including PGC‐1*α*,^[^ ^20^ ^]^ Prdm16,^[^ ^21^ ^]^ IRF4,^[^ ^22^ ^]^ Jmjd3,^[^ ^23^ ^]^ PPAR*α*,^[^ ^24^ ^]^ and Hlx.^[^ ^25^ ^]^ Notably, these factors, while powerfully activating the expression of an array of thermogenic genes, have no measurable effect on adipogenesis per se, underscoring their specialized transcriptional roles in brown and beige fat development and function. However, to date, the counterbalancing negative regulatory mechanisms are much less understood, and essential transcriptional regulators involved remain largely to be identified. A negative regulatory mechanism could be fundamental to suppressing hyperactive thermogenesis and thus sustain appropriate whole‐body homeostasis. Our present study identifies the Krüppel‐associated box (KRAB) domain‐containing protein ZFP961 as a major negative regulator and offers a deeper understanding of the transcriptional control of adipose thermogenesis.

## 2 Results

### 2.1 ZFP961 Is Enriched in Brown Adipocytes and Induced by Cold Stimulation

**Figure 1:**
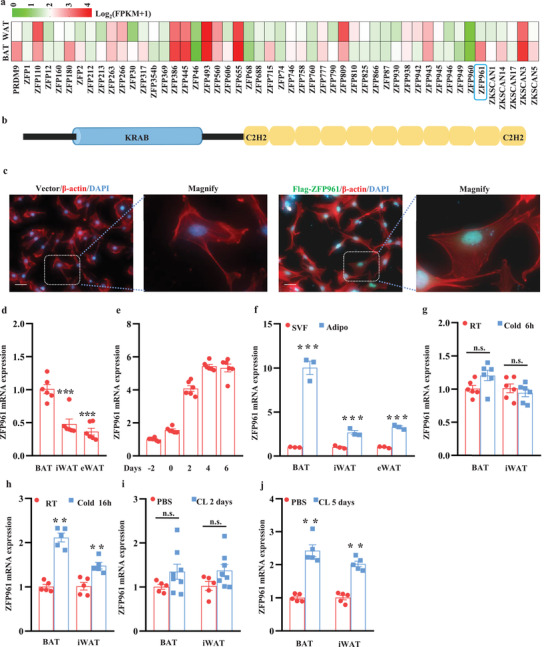
ZFP961 is BAT‐enriched and is induced by cold stimulation and *β*‐adrenergic agonist in adipose tissue. a) Relative mRNA levels in BAT versus WAT for KRAB domain‐containing zinc finger genes (*n* = 3). b) Structural domains of ZFP961. c) Immunofluorescence analysis of ZFP961 localization in immortalized brown preadipocytes. Representative pictures were shown (*n* = 3, per group). d) ZFP961 mRNA level analyzed by qRT‐PCR in adipose tissue from three‐month‐old male mice (*n* = 6, per group). e) ZFP961 mRNA level during the differentiation of immortalized brown preadipocytes (*n* = 6, per group). f) ZFP961 mRNA level in stromal vascular fraction (SVF) and mature adipocyte fraction of three‐month‐old male mice (*n* = 3, per group). g,h) ZFP961 mRNA level in BAT and iWAT of three‐month‐old male mice at cold (4 °C) for 6 h (*n* = 6, per group) and 16 h (*n* = 5, per group), respectively. i,j) ZFP961 mRNA level in BAT and iWAT of three‐month‐old male mice upon CL‐316,243 treatment for 2 d (*n* = 5–8, per group) and 5 d (*n* = 5, per group), respectively. All error bars represent s.e.m. Two‐tailed unpaired Student's *t*‐test was performed. **p < 0.01; ***p < 0.001; n.s., not significant.

KRAB domain‐containing ZFPs are potent transcriptional repressors and play widespread roles in transcriptional regulation.^[^ ^26^ ^]^ However, their biological functions in adipose tissue are unknown. We analyzed our previously published RNA‐Seq datasets (GEO: GSE56367) of mouse BAT and epididymal WAT (eWAT)^[^ ^23^ ^]^ and found that there were 50 members of KRAB domain‐containing ZFPs expressed at least in one fat depot. Among them, the ZFP961 transcript was abundantly present and had the highest enrichment in BAT compared to eWAT (**Figure** **1**a).

ZFP961 is comprised of one KRAB domain located at the N‐terminus, followed by eleven C2H2‐zinc fingers (Figure 1b). As a commercial ZFP961 antibody was unavailable, we transfected Flag‐tagged ZFP961 into immortalized brown preadipocytes to determine its localization. ZFP961 was located in the nucleus as anti‐Flag staining essentially overlapped with DAPI staining (Figure 1c). qRT‐PCR data confirmed that BAT had a much higher expression of ZFP961 than inguinal WAT (iWAT) and eWAT (Figure 1d). Moreover, ZFP961 was induced during brown adipocyte differentiation in vitro and was mainly expressed in mature adipocytes of fractionated adipose tissue (Figure 1e,f). We next evaluated ZFP961 mRNA expression upon cold stimulation. Whereas short‐term (6 h) cold challenging of C57BL/6J mice did not change ZFP961 expression (Figure 1g), cold challenging for 16 h increased ZFP961 expression in both BAT and iWAT (Figure 1h). Similarly, while there was a trend of induction of ZFP961 mRNA expression upon *β*3‐adrenergic agonist CL‐316,243 treatment for 2 d (Figure 1i), treatment of the mice for 5 d markedly increased its expression in both BAT and iWAT (Figure 1j). Given ZFP961 as a potential transcriptional repressor, these results raised the idea that ZFP961 might act to limit thermogenic gene expression to prevent energy over‐consumption in adipose tissue, especially during prolonged stimulation of thermogenesis.

### 2.2 ZFP961 Suppresses Thermogenic Activation in Adipocytes

**Figure 2:**
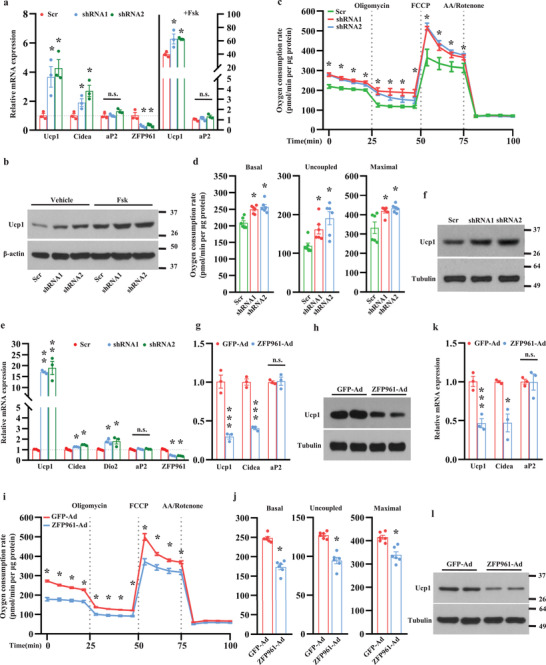
ZFP961 blocks thermogenic gene expression in vitro. a) Gene expression in ZFP961 knockdown mature brown adipocytes that were treated with or without forskolin (Fsk, 10 µm) for 6 h (*n* = 3, per group). b) Ucp1 protein in ZFP961 knockdown mature brown adipocytes that were treated with or without Fsk (10 µm) for 12h. c) Oxygen consumption rate (OCR) in ZFP961 knockdown mature brown adipocytes (*n* = 6, per group). Cells were treated with oligomycin, FCCP, and antimycin (AA)/rotenone at the indicated time points. d) The average basal, uncoupled, and maximal respiration rates were shown (*n* = 6, per group). e) Primary iWAT preadipocytes were infected with lentivirus containing ZFP961 shRNAs or Scr shRNA and differentiated. Gene expression was analyzed by qRT‐PCR (*n* = 3, per group). f) Ucp1 protein in primary iWAT adipocytes generated as in (e). g) Gene expression in mature brown adipocytes infected with adenovirus expressing ZFP961 or GFP (*n* = 3, per group). h) Ucp1 protein in brown adipocytes generated as in (g). i) OCR analysis in brown adipocytes generated as in (g) (*n* = 6, per group). j) The average basal, uncoupled, and maximal respiration rates were shown (*n* = 6, per group). k) In vitro differentiated primary iWAT adipocytes were infected with adenovirus expressing ZFP961 or GFP. Gene expression was assessed by qRT‐PCR (*n* = 3). l) Ucp1 protein in primary iWAT adipocytes generated as in (k). All error bars represent s.e.m. Two‐tailed unpaired Student's *t*‐test was performed. *p < 0.05; **p < 0.01; ***p < 0.001; n.s., not significant.

To investigate whether ZFP961 is engaged in adipose thermogenesis, we used lentivirus to stably express two ZFP961 knockdown constructs (shRNAs) in immortalized brown preadipocytes, which were then induced to mature adipocytes. As shown in **Figure** **2**a, ZFP961 shRNAs strongly reduced ZFP961 mRNA expression but did not affect adipocyte differentiation as indicated by Oil Red O staining, C/EBP*α* immunostaining (Figure S1a,b, upper panel, Supporting Information), and the expression level of the common fat gene aP2 (Figure 2a). Strikingly, Ucp1 and Cidea mRNA (Figure 2a) and Ucp1 protein (Figure 2b and Figure S1b, lower panel, Supporting Information) levels were increased. Silencing of ZFP961 further elevated forskolin‐induced Ucp1 mRNA and protein expression (Figure 2a, right panel, and Figure 2b). Moreover, mitochondrial respiration and oxygen consumption rate were increased in brown adipocytes (Figure 2c,d); however, the relative contribution of Ucp1 to this increase requires further study. Similar to brown adipocytes, downregulation of ZFP961 in primary adipocytes isolated from iWAT did not affect adipogenesis (Figure 2e and Figure S1c,d, upper panel, Supporting Information) but increased Ucp1, Cidea, and Dio2 mRNA (Figure 2e) and Ucp1 protein expression levels (Figure 2f and Figure S1d, lower panel, Supporting Information).

To complement these loss‐of‐functional studies, we generated adenovirus to overexpress ZFP961 in differentiated adipocytes. Ectopic expression of ZFP961 in brown adipocytes decreased Ucp1 and Cidea mRNA (Figure 2g) and Ucp1 protein levels (Figure 2h and Figure S1f, lower panel, Supporting Information) without any effect on adipogenesis (Figure 2g and Figure S1e,f, upper panel, and Figure S1g, Supporting Information). Likewise, ectopic expression of ZFP961 reduced mitochondrial respiration and oxygen consumption rate (Figure 2i,j). We observed similar results in primary iWAT cells (Figure 2k,l and Figure S1h–j, Supporting Information). Collectively, these results show that ZFP961 potently represses adipocyte thermogenesis in vitro.

### 2.3 Acute Down‐ and Up‐regulation of ZFP961 via Fat‐Specific Viral Injection Remodels iWAT In Vivo

**Figure 3:**
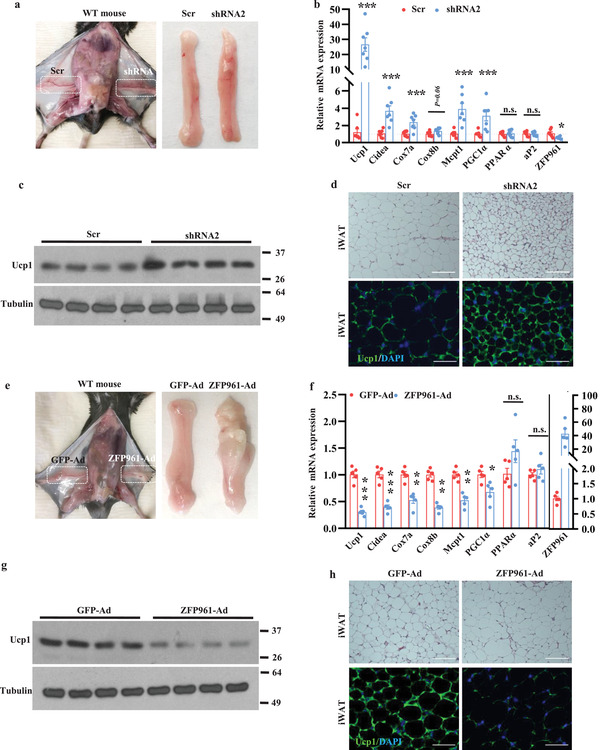
Down‐ and up‐regulation of ZFP961 remodels iWAT in vivo. a) Representative image of wild‐type male mice injected with lentivirus containing ZFP961‐shRNA2 or Scr‐shRNA to show gross iWAT appearance. b) Gene expression in iWAT of mice generated as in (a) (*n* = 7, per group). c) Ucp1 protein in iWAT of mice generated as in (a) (*n* = 4 per group). d) H&E staining and immunostaining of Ucp1 in iWAT of mice generated as in (a). Representative images were shown (*n* = 3, per group). Scale bar = 200 µm. e) Representative image of wild‐type male mice injected with adenovirus containing ZFP961 or GFP to show gross iWAT appearance. f) Gene expression in iWAT of mice generated as in (e) (*n* = 5, per group). g) Ucp1 protein in iWAT of mice generated as in (e) (*n* = 4, per group). h) H&E staining and immunostaining of Ucp1 in iWAT of mice generated as in (e). Representative images were shown (*n* = 3, per group). Scale bar = 200 µm. All error bars represent s.e.m. Two‐tailed unpaired Student's *t*‐test was performed. *p < 0.05; **p < 0.01; ***p < 0.001; n.s., not significant.

We next investigated whether ZFP961 suppresses the thermogenic program in adipose tissue in vivo. We purified high‐titer lentiviruses expressing ZFP961‐shRNA and scramble control, and injected them into the left and right iWAT pads of the same animal, respectively. One week after injection, we dissected the mice. On visual inspection, iWAT pads injected with ZFP961‐shRNAs were evidently redder than those injected with scramble control (**Figure** **3**a). Strikingly, acute silencing of ZFP961 markedly elevated the expression of Ucp1 (26‐fold), Cidea, and PGC‐1*α*, and genes encoding mitochondrial enzymes (Cox7a and Mcpt1) (Figure 3b,c), but the expression of the common fat gene aP2 was unchanged (Figure 3b). Similar results were obtained with a second ZFP961‐shRNA construct (Figure S2, Supporting Information). Histological analysis showed that upon ZFP961 knockdown, adipocyte size and lipid droplet size were smaller, and immunofluorescence staining revealed that these adipocytes were almost all Ucp1‐positive (Figure 3d). These results demonstrate that acute downregulation of ZFP961 results in a robust iWAT browning.

We then asked if ectopic expression of ZFP961 in vivo could negatively control adipose thermogenic gene expression. We performed similar experiments as above using adenoviruses expressing ZFP961 and GFP. One week after injection, we dissected the mice and found that iWAT pads injected with ZFP961‐ad appeared whiter (Figure 3e). As shown in Figure 3f,g, overexpression of ZFP961 dramatically reduced the expression of Ucp1, Cidea, Cox7a, Cox8b, Mcpt1, and PGC‐1*α*, but not ap2 gene expression (Figure 3f). As might be expected, the lipid droplet was more extensive, and levels of Ucp1 immunostaining were significantly diminished (Figure 3h). Thus, acute down‐ and up‐regulation of ZFP961 through viral delivery directly led to remarkable but opposite remodeling of iWAT in vivo. Collectively, these results uncover ZFP961 as a critical negative regulator of the adipose thermogenic program in vivo.

### 2.4 ZFP961 Knockout Mice Display Increased Adipose Thermogenesis and Energy Expenditure

**Figure 4:**
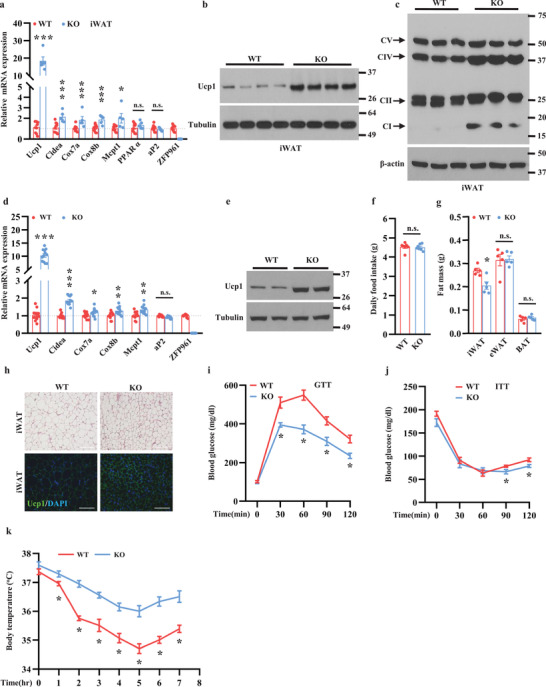
Ablation of ZFP961 promotes adipose thermogenesis and improves cold tolerance in vivo. a) Gene expression in iWAT of ten‐week‐old male KO mice (*n* = 5, per group) and littermate control male mice (*n* = 10, per group). b) Ucp1 protein in iWAT of ten‐week‐old male KO mice and littermate control male mice (*n* = 4, per group). c) OXPHOS protein expression in iWAT of ten‐week‐old male KO and littermate control male mice (*n* = 3, per group). d) Gene expression in primary iWAT cells isolated from two‐week‐old male KO mice and littermate control male mice (*n* = 12, per group). e) Ucp1 protein expression in primary iWAT cells generated in (d). f) Daily food consumption of normal chow in KO male mice and littermate control male mice (*n* = 7, per group). g) Fat mass of 12‐week‐old male KO mice and littermate control male mice (*n* = 5, per group). h) H&E staining and immunostaining of Ucp1 in iWAT of 12‐week‐old male KO mice and littermate control male mice. Representative images were shown (*n* = 3, per group). Scale bar = 200 µm. i,j) Glucose tolerance test and insulin tolerance test of 12‐month‐old male KO mice and littermate control male mice on normal chow (*n* = 7, per group), respectively. The mice were fasted for 16 and 6 h, respectively. k) Rectal core body temperatures of 16‐week‐old male KO mice (*n* = 10, per group) and littermate control male mice (*n* = 9, per group) upon cold stimulation at indicated time points. All error bars represent s.e.m. Two‐tailed unpaired Student's *t*‐test was performed. *p < 0.05; **p < 0.01; ***p < 0.001; n.s., not significant.

We used the whole body ZFP961 knockout (KO) mice to investigate the physiological importance of this gene in adipose thermogenesis. BAT of KO mice showed a very slight increase of mRNA levels of Ucp1, Cox8b, and Mcpt1 (Figure S3a, Supporting Information), and Ucp1 protein level was almost no change (Figure S3b, Supporting Information). Similar results were obtained from female mice (Figure S3c, Supporting Information). On the other hand, iWAT of the KO mice displayed robust increases of Ucp1 (18‐fold) and Cidea mRNA levels (**Figure** **4**a) and Ucp1 protein levels (Figure 4b) compared with that of littermate control mice. Genes for mitochondrial metabolism, such as Cox7a, Cox8b, and Mcpt1, were also increased. Similar results were obtained from female mice (Figure S3d, Supporting Information). Moreover, levels of mitochondrial oxidative phosphorylation (OXPHOS) complexes were increased in iWAT from KO mice (Figure 4c) but were unchanged in BAT (Figure S3e, Supporting Information). Notably, the expression of Ucp1, Cidea, Cox7a, Cox8b, and Mcpt1 was increased in in vitro differentiated iWAT culture isolated from KO mice, while expression of the common fat gene aP2 was unchanged (Figure 4d,e), suggesting that iWAT browning of KO mice is an adipose‐autonomous effect.

We did not see any difference in food intake between KO and control mice under the regular chow diet (Figure 4f). While the BAT mass and epididymal WAT mass remained unchanged, the inguinal WAT mass in KO mice was significantly decreased compared to control mice (Figure 4g), most likely due to increased adipose energy expenditure. Consistent with this result, iWAT adipocytes of KO mice were much smaller in size, and they were almost all Ucp1‐positive (Figure 4h), whereas there was no difference in BAT or eWAT (Figure S3f, Supporting Information). We then performed glucose and insulin tolerance tests. While no difference was observed in young animals, aged KO mice were more glucose tolerant and had lower glucose levels in late time points during insulin tolerance test (Figure 4i,j), consistent with the idea that iWAT browning improves glucose homeostasis, especially in aged mice.^[^ ^2^, ^27^, ^28^ ^]^ In a cold challenge experiment, the core body temperatures of the KO mice were significantly higher than control mice (Figure 4k). Similar results were obtained from female mice (Figure S3g, Supporting Information). Altogether, our findings indicate that ZFP961 is a physiological brake for adipose thermogenesis to maintain adiposity and energy balance.

### 2.5 Reexpression of ZFP961, But Not the KRAB Domain Deletion Mutant, Blocks the iWAT Browning in ZFP961 Knockout Mice

**Figure 5:**
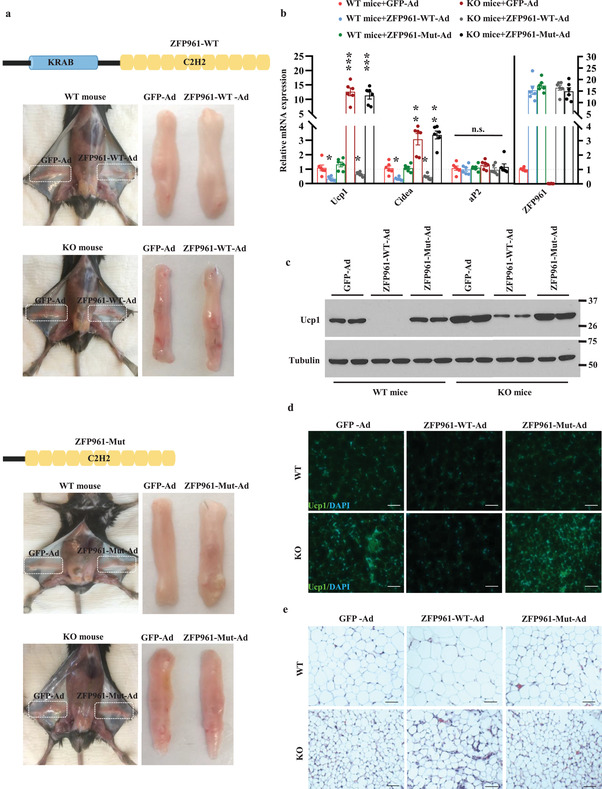
Adipose‐specific reexpression of ZFP961 rescues phenotypes of ZFP961‐KO mice. a) Representative image of WT and KO mice injected with adenovirus expressing ZFP961‐WT, ZFP961‐Mut, or GFP to show gross iWAT appearance. b) Gene expression in iWAT of mice as in (a) (*n* = 6, per group). c) Ucp1 protein in iWAT of mice as in (a) (*n* = 2, per group). d,e) immunostaining of Ucp1 and H&E staining in iWAT of mice as in (a), respectively. Representative images were shown (*n* = 3, per group). Green = Ucp1; Blue = DAPI; Scale bar = 100 µm. All error bars represent s.e.m. Two‐tailed unpaired Student's *t*‐test was performed. *p < 0.05; **p < 0.01; ***p < 0.001; n.s., not significant.

To definitively validate that the increased thermogenic gene expression in ZFP961 knockout mice was indeed caused by loss of ZFP961 in adipose tissue, and to assess the importance of the KRAB domain, we performed the rescue experiments by injection of adenoviruses expressing full‐length ZFP961 (ZFP961‐WT) or KRAB domain deletion mutant (ZFP961‐Mut) into iWAT depots. While iWAT pads injected with ZFP961‐WT‐ad appeared whiter compared with the ones injected with GFP‐ad in both WT and KO mice (Figure 3e, and **Figure** **5**a, upper panels), this was not observed in iWAT pads injected with ZFP961‐Mut‐ad (Figure 5a, lower panels). Restoration of ZFP961‐WT in iWAT markedly suppressed the induction of Ucp1 and Cidea (Figure 5b,c) expression caused by ZFP961 deficiency, whereas the expression of common fat gene aP2 was unchanged (Figure 5b). Importantly, overexpression of ZFP961‐Mut had no inhibitory effect on thermogenic gene expression (Figure 5b) and Ucp1 protein expression (Figure 5c) in both WT and KO mice, despite that similar mRNA level of mutant and full‐length ZFP961 were produced (Figure 5b). Immunostaining showed that restoration of ZFP961‐WT in iWAT negated the increased formation of Ucp1‐positive adipocytes observed in KO mice, whereas the ZFP961‐Mut failed to decrease the number of Ucp1‐positive adipocytes in both WT and KO mice (Figure 5d). Furthermore, the iWAT adipocyte size of the KO mice with ZFP961‐WT injection was restored to size similar as in WT mice but remained smaller with ZFP961‐Mut injection (Figure 5e). These results suggest that the iWAT browning phenotype in KO mice is due to loss of ZFP961 in adipose tissue and that the KRAB domain of ZFP961 is required for suppression of thermogenic gene expression.

### 2.6 Ablation of ZFP961 Protects Mice against Diet‐Induced Obesity

**Figure 6:**
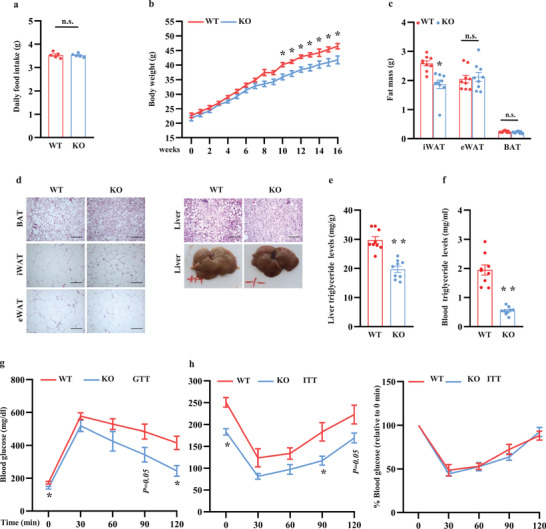
ZFP961‐KO mice are protected from diet‐induced obesity and metabolic dysfunction. a) Daily food consumption of high‐fat diet in KO male mice and littermate control male mice (*n* = 5, per group). b) Weekly body weight of KO male mice and littermate control male mice on a high‐fat diet (*n* = 9, per group). c) Fat mass of KO male mice and littermate control male mice on a high‐fat diet (*n* = 9 per group). d) H&E staining of BAT, iWAT, eWAT, and liver of high‐fat diet fed KO male mice and littermate control male mice. Representative images were shown (*n* = 3, per group). Scale bar = 100 µm. e,f) Hepatic and circulating triglyceride levels of KO male mice and littermate control male mice on a high‐fat diet (*n* = 9, per group), respectively. g,h) Glucose tolerance test and insulin tolerance test of KO male mice and littermate control male mice on a high‐fat diet (*n* = 5, per group). The mice were fasted for 16 and 6 h, respectively. All error bars represent s.e.m. Two‐tailed unpaired Student's *t*‐test was performed. *p < 0.05; **p < 0.01; n.s., not significant.

We next analyzed the beneficial effects associated with increased thermogenesis at conditions of energy overload. The KO mice and littermate control mice were challenged with a high‐fat diet (HFD, 60% calorie). There was no difference in daily food intake (**Figure** **6**a), which was measured in individually caged mice. Body weight was then monitored in a separate cohort of group‐housed mice. We found that KO mice gained 5 g less body weight compared to littermate control mice (Figure 6b). While BAT and eWAT mass were unchanged, iWAT fat mass was significantly decreased (Figure 6c), consistent with the elevation of thermogenic gene expression in this fat depot. Adipocytes of KO mice were smaller in size (Figure 6d). Loss of ZFP961 significantly ameliorated HFD‐induced liver steatosis (Figure 6d, right panel, bottom, and Figure 6e) and lowered circulating triglyceride accumulation (Figure 6f). The KO mice were also more glucose tolerant and had decreased basal glucose level (Figure 6g,h). Please note, glucose and insulin were dosed based on body weight, which may potentially worsen glucose intolerance but mask insulin intolerance in wild type mice. Altogether, these findings indicate that upregulation of adipose thermogenesis by loss of ZFP961 prevents mice from diet‐induced obesity, hepatic steatosis, and hyperglycemia.

### 2.7 ZFP961 Coordinates with PPAR*α* to Exert Thermogenic Gene Repression

**Figure 7:**
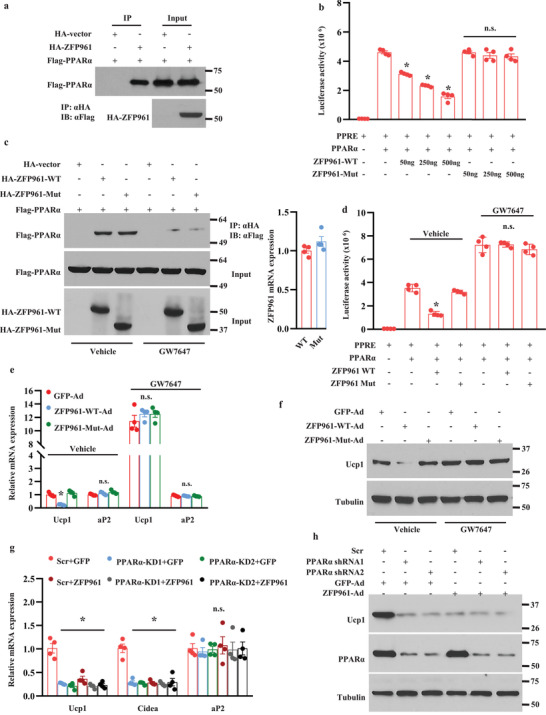
ZFP961 interacts with PPAR*α* to block thermogenic activation. a) Co‐immunoprecipitation analysis using cell extracts from HEK293T cells that were co‐transfected with HA‐ZFP961 and Flag‐PPAR*α*. b) HEK293T cells were co‐transfected with indicated plasmids, and the transcriptional activity of PPAR*α* was measured with luciferase reporter assay (*n* = 4, per group). c) Left panel: co‐immunoprecipitation analysis using cell extracts from HEK293T cells that were co‐transfected with HA‐ZFP961‐WT, HA‐ZFP961‐Mut, or Flag‐PPAR*α* in the presence or absence of GW7647 (1 × 10^−6^ m). Right panel: ZFP961‐Mut and ZFP961 mRNA levels were analyzed by qRT‐PCR (*n* = 4, per group). d) The transcriptional activity of PPAR*α* was measured in HEK293T cells that were co‐transfected with indicated plasmids and treated with or without GW7647 (1 × 10^−6^ m) (*n* = 4, per group). e) Mature brown adipocytes were infected with adenovirus expressing ZFP961‐WT, ZFP961‐Mut, or GFP and then treated with or without GW7647 (1 × 10^−6^ m) for 24 h. Gene expression was analyzed (*n* = 4, per group). f) Ucp1 protein in mature brown adipocytes generated as in (e). g) Brown preadipocytes infected with lentivirus containing Scr or PPAR*α* shRNAs were differentiated and then infected with adenovirus expressing ZFP961‐WT or GFP, and gene expression was analyzed (*n* = 4, per group). h) Ucp1 protein in mature brown adipocytes generated as in (g). All error bars represent s.e.m. Two‐tailed unpaired Student's *t*‐test was performed. *p < 0.05; n.s., not significant.

Activation of thermogenic gene expression is orchestrated by a number of critical transcriptional regulators, such as PGC1*α*,^[^ ^20^ ^]^ PRDM16,^[^ ^21^ ^]^ PPAR*α*,^[^ ^24^ ^]^ and Hlx.^[^ ^25^ ^]^ We thus sought to determine whether ZFP961 suppresses the activity of any of these factors. We performed co‐immunoprecipitation to examine their interaction. We observed a strong interaction of ZFP961 with PPAR*α* in HEK293T cells (**Figure** **7**a), whereas there was no interaction with Hlx, PGC1*α*, or PRDM16 (Figure S5a–c, Supporting Information). In a luciferase reporter assay, ZFP961 dose‐dependently decreased PPAR*α* transcriptional activity, while ZFP961‐Mut, which lacks the KRAB domain, had no effect (Figure 7b), although ZFP961‐Mut still retained its interaction with PPAR*α* (Figure 7c). Of note, ZFP961‐Mut protein was as stable as wild type ZFP961 protein and similar amounts of protein were produced in cells transfected with same amounts of plasmids. Interestingly, in the presence of PPAR*α* agonist GW7647, ZFP961 was no longer able to interact with PPAR*α* (Figure 7c) and to suppress the luciferase reporter activity of PPAR*α* (Figure 7d). Consistent with these observations, while adenoviral expression of full‐length ZFP961, but not ZFP961‐Mut, inhibited the expression of Ucp1 mRNA and protein in cultured brown adipocytes, this inhibition was abolished by PPAR*α* agonist GW7647 (Figure 7e,f). To further confirm that repression of thermogenic gene expression by ZFP961 was mediated via PPAR*α*, we expressed ZFP961 in PPAR*α*‐depletion mature adipocytes. As shown in Figure 7g,h, overexpression of ZFP961 did not further reduce Ucp1 and Cidea mRNA expression and Ucp1 protein level in PPAR*α*‐depletion adipocytes compared with PPAR*α* knockdown alone. These results collectively suggest that ZFP961 interacts with PPAR*α* to repress the thermogenic gene program. Of note, ZFP961 does not repress the expression of PPAR*α* (Figures 3b,f and 4a).

## 3 Discussion

The transcriptional mechanisms that activate adipose thermogenesis have been extensively investigated, and a number of key transcriptional regulators have been identified.^[^ ^18^ ^]^ These studies have elucidated a multilayer, delicate network that specifically induces the expression of BAT‐selective genes. In contrast, the counterbalancing negative regulatory mechanisms remain obscure. In the current study, using both in vitro and in vivo gain‐of‐function and loss‐of‐function approaches, we demonstrate that the KRAB domain‐containing Zinc finger protein ZFP961 serves as a physiological rheostat that represses thermogenic gene expression program through coordinating with PPAR*α*. While our present work focuses on the role of ZFP961 at homeostatic state, it will be interesting in future studies to determine whether ZFP961 plays a part in curbing thermogenesis at thermic conditions such as infection, cancer cachexia, burn injury, and hyperthyroidism, in which energy‐wasting is unwanted.

Our data show that the KRAB domain of ZFP961 is required for repressing iWAT browning. Given that KRAB domain‐containing ZFPs are a huge family, we anticipate that additional members likely participate in thermogenic regulation as well. It is also important to note that, to date, little study has been done to address the metabolic functions of these KRAB domain‐containing ZFPs in metabolically active tissues. Our study illustrates a paradigm that a transcriptional regulator uses its repression domain to exert powerful negative control of metabolic physiology, highlighting the necessity for this area of research with KRAB‐ZFPs.

While our knockout studies were performed in ZFP961 null mice, we believe that the iWAT browning phenotype is adipose‐autonomous, as supported by data from in vitro primary adipocyte culture isolated from KO mice and in vivo rescue experiments. iWAT browning typically leads to improvement of glucose homeostasis and amelioration of HFD‐induced obesity and hepatic steatosis; this is precisely what we observed in KO mice. Although we cannot rule out the contribution of other tissues, the robust iWAT browning is at least partially responsible for the metabolic benefits in the KO mice.

ZFP961 is induced by long‐term cold and *β*3‐adrenergic stimulation in both BAT and iWAT. Moreover, ZFP961 is highly enriched in BAT compared with WAT at ambient temperature, probably due to relatively higher basal constitutive *β*3‐adrenergic signaling. However, loss of ZFP961 has no appreciable effect on BAT. Instead, the thermogenic program in iWAT is unleashed. On the basis of the great mass of iWAT depot and its high recruitability by cold and adrenergic signaling, it appears logical that iWAT utilizes ZFP961 as a built‐in feedback safety net to prevent energy over‐consumption during prolonged thermogenesis.

Interestingly, PPAR*α* is BAT‐enriched and is induced by cold and *β*‐adrenergic agonist stimulation, a similar expression pattern as that of ZFP961 (Figure S6a–i, Supporting Information). Our studies suggest that ZFP961 is recruited by PPAR*α* to repress thermogenesis. In the presence of PPAR*α* agonist, ZFP961 is released from the PPAR*α* complex and is no longer able to inhibit thermogenic gene expression. As PGC‐1*α* co‐activates PPAR*α* to enhance thermogenesis,^[^ ^29^ ^]^ our data indicate a possible competition or exchange between PGC‐1*α* and ZFP961 to regulate PPAR*α* thermogenic function. Another interesting aspect is that, as a repressor, ZFP961 does not regulate adipogenesis per se. Moreover, since ZFP961 is BAT‐enriched and is induced by cold and *β*3‐adrenergic stimulation, we initially considered the possibility that ZFP961 might repress WAT‐selective gene expression in BAT and beige fat. However, this was not observed (Figure S4a,b, Supporting Information); instead, we observed the opposite: repression of BAT‐selective genes. These results, although somewhat surprising, suggest a very specialized role of ZFP961 in adipose tissue and are consistent with observations that PPAR*α* does not regulate general adipogenesis or expression of WAT‐selective genes (Figure S5d,e, Supporting Information).

In summary, we identify ZFP961/PPAR*α* axis as a physiological and powerful brake system to safeguard adipose thermogenesis. Our work expands our understanding of the complexity of regulatory networks in thermogenic adipose tissue and provides new insights into the negative feedback mechanisms that sustain whole‐body energy balance and tissue homeostasis.

## 4 Experimental Section

### Animal Studies

*ZFP961* knockout mice in a C57BL/6 background (Stock No. 024057) and C57BL/6 wild‐type mice (Stock No. 000664) were purchased from Jackson Laboratory. All animals were housed in a 12‐h‐light/12‐h‐dark cycle at a temperature‐controlled (23 °C ± 0.9 °C) facility with free access to food and water. The mice were fed with a regular diet containing 4% (w w^−1^) fat or a high‐fat diet (Bioserv, cat. no. S3282) containing 36% (w w^−1^) fat. For the food intake experiment, daily food consumption was measured for individually caged mice. For the glucose tolerance test, the mice were intraperitoneally (IP) injected with glucose at 2 g kg^−1^ body weight after 16 h fasting. For the insulin tolerance test, the mice were IP injected with insulin at 0.75 U kg^−1^ body weight after 6 h fasting. For the cold challenging test, the mice were individually caged with food withdrawn and water provided, placed in a 4 °C cold room, and core body temperature was detected with Microtherma 2 rectal probe (Thermoworks). Gender‐matched littermate controls were used in all the experiments with age indicated. All animal studies were performed according to procedures approved by the University of Massachusetts Medical School's Institutional Animal Care and Use Committee (IACUC).

### Cell Culture, Differentiation, and Treatments

The immortalized brown preadipocyte cell line was generated previously.^[^ ^30^ ^]^ At day ‐2 of differentiation, the medium containing 20 × 10^−9^ m insulin and 1 × 10^−9^ m 3,3′,5‐triiodo‐l‐thyronine (differentiation medium) was added into brown preadipocytes at 70% confluence. On day 0, differentiation was induced by culturing in the differentiation medium containing 0.125 × 10^−3^ m indomethacin, 0.5 × 10^−6^ m dexamethasone, and 0.5 × 10^−3^ m isobutylmethylxanthine for 48 h. After the induction period, cells were changed back to the differentiation medium. Primary iWAT preadipocyte culture and differentiation were described previously.^[^ ^25^ ^]^ Preadipocytes were isolated from two‐week‐old mice. Differentiation was started by culturing confluent cells in DMEM/F12 (Gibco) medium containing 10% FBS, 850 × 10^−9^ m insulin, 1 × 10^−9^ m triiodothyronine, 0.5 × 10^−3^ m isobutylmethylxanthine, 0.5 × 10^−6^ m dexamethasone, and 0.125 × 10^−3^ m indomethacin. After 2 d, cells were cultured in DMEM/F12 medium containing 10% FBS, 850 × 10^−9^ m insulin, and 1 × 10^−9^ m triiodothyronine, and cell medium was changed every 2 d. On day 6, both brown and primary iWAT adipocytes were fully differentiated, and cells with at least 95% differentiation efficiency were used in experiments. In some experiments, differentiated adipocytes were treated with forskolin (10 × 10^−6^ m) (Acros Organics, cat. no. BP2520), as indicated.

### Plasmids and Viruses

All plasmids were verified by sequencing. *ZFP961* overexpression and knockdown adenoviruses were generated using the pAdEasy‐1 system, and viruses were purified as described previously.^[^ ^25^ ^]^ *ZFP961* and *PPARα* shRNA knockdown lentiviral constructs were generated using PSP‐108 vector ^[^ ^31^ ^]^ (Addgene), and viruses were produced by co‐transfection along with helper plasmids pMD2.G (Addgene) and psPAX2 (Addgene) into HEK293T cells. All viral titers were predetermined, and the same number of viral particles was used in control and experimental samples. *ZFP961* knockdown targeting sequences are: shRNA‐1, 5′ GGGAAAGCATTCATGAGAT 3′; shRNA‐2, 5′ CACACTACATCTAACTCTA 3′. *PPARα* knockdown targeting sequences are: shRNA‐1, 5′ GCTATGAAGTTCAATGCCTTA 3′; shRNA‐2, 5′ CCAACGGCGTCGAAGACAA 3′.

### In Vivo Lentivirus and Adenovirus Injection

The in vivo lentivirus and adenovirus injection was performed as previously described.^[^ ^25^ ^]^ An equal number of experimental viruses and control viruses were injected into the left and right iWAT pad of the same animal, respectively. High‐titer lentiviruses (1 × 10^9^ transducing unit mL^−1^) were injected into four different spots of iWAT at 50 µL per spot/injection to cover the whole fat pad. Adenoviruses were injected into the center of iWAT pads within a marked area of ≈0.5 cm in diameter at 1 × 10^10^ pfu per injection. On day 8, the mice were sacrificed, and the whole iWAT fat pads (knockdown) or iWAT tissues from the injected area (overexpression) were harvested for gene expression, Western blotting, and histology.

### Histology and Immunofluorescence

For H&E staining, tissues were fixed with 10% formalin and paraffin‐embedded. The H&E staining was performed by standard procedures. For immunofluorescence staining, the formalin‐fixed and paraffin‐embedded tissue sections were deparaffinized and rehydrated by graded concentrations of ethanol solutions. After pre‐incubation with blocking buffer (PBS containing 5% normal goat serum and 0.3% Triton X‐100) for 60 min at room temperature, the slides were incubated with Ucp1 antibody (Sigma, cat. no. U6382) (1:200 dilution) in blocking buffer at 4 °C overnight. Next, the slides were washed and incubated with Alexa Fluor 488‐conjugated secondary antibody (Thermo Fisher, cat. no. A‐11034) and DAPI (Sigma, cat. no. D9542) for 2 h at room temperature. Images were acquired and processed with the same settings. For in vitro Ucp1 and CEBP/*α* staining, mature adipocytes were fixed with 3.7% formalin at 37 °C for 15 min and gently rinsed twice with PBS. Cells were permeabilized with ice‐cold 100% methanol at −20 °C for 10 min and washed twice with PBS. Subsequently, the cells were incubated with blocking buffer (PBS containing 5% normal goat serum and 0.3% Triton X‐100) for 60 min at room temperature and incubated with Ucp1 antibody (Sigma, cat. no. U6382) (1:200 dilution) and CEBP/*α* antibody (Santa Cruz, cat. no. sc‐61) (1:100 dilution) in blocking buffer at 4 °C overnight. After incubation, the cells were gently washed twice with PBS and incubated with Alexa Fluor 488‐conjugated (Thermo Fisher, cat. no. A‐11034) or 594‐conjugated secondary antibody (Thermo Fisher, cat. no. A‐11011) for 2 h at room temperature. Finally, the cells were incubated with DAPI (Sigma, cat. no. D9542) for 60 min at room temperature, and images were acquired with a fluorescence microscope.

### Oil Red O Staining

The differentiated mature adipocytes at day 6 in six‐well plates were gently washed twice with PBS and fixed with 3.7% paraformaldehyde for 20 min at room temperature. Subsequently, cells were stained with filtered Oil Red O (Sigma, cat. no. O0625) for 2 h, and then the stained cells were gently washed with distilled water three times before visualization.

### Cellular Respiration Analysis

The immortalized brown preadipocytes were first infected with lentivirus containing scramble control or ZFP961 shRNAs and selected with puromycin. Cells were then trypsinized, and equal numbers of cells were seeded on XF96 Cell Culture Microplates and differentiated. For overexpression experiments, equal numbers of brown preadipocytes were cultured in the XF96 Cell Culture Microplates and were infected with adenovirus containing GFP control or ZFP961 at differentiation day 4. Oxygen consumption rate (OCR) was assessed on day 6 by an XF96 Extracellular Flux Analyzer (Seahorse Bioscience). The uncoupled and maximal OCR was determined using oligomycin (Sigma, cat. no. O‐4876, 4 × 10^−6^ m) and FCCP (Sigma, cat. no. C‐2920, 4 × 10^−6^ m), respectively. The complex III and complex I‐dependent respiration were blocked by Antimycin A (Sigma, cat. no. A8674, 2 × 10^−6^ m) and rotenone (Sigma, cat. no. R8875, 2 × 10^−6^ m), respectively.

### Luciferase Reporter Assay

HEK293T cells were cultured in 24‐well plates. The cells were transfected with PPRE luciferase reporter plasmid and PPAR*α* plasmid along with ZFP961‐WT or ZFP961‐Mut plasmids using Lipofectamine 2000 (Invitrogen, cat. no. 11668‐019). Vector plasmid was used to adjust the total amount of plasmids for each well. In some experiments, cells were treated with GW7647 (1 × 10^−6^ m) (Santa Cruz Biotechnology, cat. no. CAS 265129‐71‐3) as indicated. Luciferase activity was measured 48 h after transfection.

### Western Blotting and Co‐immunoprecipitation

Western blotting was carried out as described previously.^[^ ^32^ ^]^ Equal amounts of adipose tissues or cultured mature adipocytes were homogenized with lysis buffer [100 × 10^−3^ m NaCl, 50 × 10^−3^ m Tris (pH 7.5), 0.5% Triton X‐100, 5% (w v^−1^) glycerol] containing protease inhibitor cocktail (Complete Mini‐EDTA free, Roche, cat. no. 11836170001). The lysates were centrifuged at 13 000 rpm for 10 min at 4 °C. Equal amounts of protein were separated by SDS‐PAGE, transferred to PVDF membrane, and immunoblotted with primary antibodies against Ucp1 (Sigma, cat. no. U6382), total OXPHOS Rodent WB Antibody Cocktail (Abcam, cat. no. ab110413), PPAR*α* (Santa Cruz Biotechnology, cat. no. sc‐398394), Tubulin (DSHB, cat. no. E7‐S), *β*‐actin (Santa Cruz Biotechnology, cat. no. sc‐47778), HA‐tag (BioLegend, cat. no. 901502), and Flag‐Tag (Sigma, cat. no. F7425). To examine the interaction between ZFP961 and PPAR*α*, Hlx, PGC‐1*α*, or PRDM16, HA‐ZFP961 or HA vector along with Flag‐PPAR*α*, Flag‐Hlx, Flag‐PGC‐1*α*, or Flag‐PRDM16 were co‐transfected into HEK293 cells. After 48 h, cell extracts were generated in the above lysis buffer and incubated for 2 h with anti‐HA agarose beads (Santa Cruz Biotechnology, cat. no. sc‐7392 AC). After incubation, the beads were washed with washing buffer [100 × 10^−3^ m NaCl, 50 × 10^−3^ m Tris (pH 7.5), 0.1% NP‐40, 3% glycerol], and immunoprecipitates were analyzed by Western blotting.

### Gene Expression Analysis

Total RNA was extracted from mature adipocytes or tissues using TRIzol reagent (Invitrogen, cat. no. 15596‐018) according to the manufacturer's instructions, and an equal amount of RNA was used to perform the reverse transcription. Quantitative real‐time PCR (qRT‐PCR) was carried out with SYBR green fluorescent Dye (Bio‐Rad, cat. no. 1725272) using an ABI7300 PCR instrument, and ribosomal 36B4 (U36) was served as an internal control. The relative mRNA expression level was calculated by the ΔΔ‐Ct method. Primer sequences are available upon request.

### Statistical Analysis

The sample size was determined by experience and based on similar work in the literature. The investigators who performed mouse experiments were not blinded to genotypes. Statistical analyses and the number of biological samples (*n*) were described in detail for each figure panel. Unless otherwise stated, data were presented as mean ± standard error of the mean (s.e.m.), and individual data points were plotted. GraphPad Prism 8.0 software was used for all statistical analysis. Statistical analysis was performed using a two‐tailed Student's *t*‐test. Statistical significance was considered as *P* <0.05 and expressed as *P < 0.05, **P < 0.01, ***P < 0.001.

## Conflict of Interest

The authors declare no conflict of interest.

## Author Contributions

L.H. and Q.Y.Y. designed and performed the experiments and analyzed the data. P.P.L. analyzed the bioinformatics data. Y.‐X.W. designed the experiments and analyzed the data. L.H. and Y.‐X.W. wrote the manuscript.

## Supporting information

Supporting Information

## Data Availability

The data that support the findings of this study are available in the Supporting Information.
