# Supplementary material for: The KRAB Domain‐Containing Protein ZFP961 Represses Adipose Thermogenesis and Energy Expenditure through Interaction with PPARα
Source: Adv Sci (Weinh). 2021 Nov 7;9(2):2102949. doi: 10.1002/advs.202102949 (PMC8805557; doi:10.1002/advs.202102949)
Supplement: Supplementary file 1 — Supporting Information [file ADVS-9-2102949-s001.pdf]

ADVANCED SCIENCE
Open Access

Supporting Information

for *Adv. Sci.*, DOI: 10.1002/advs.202102949

The KRAB Domain-containing Protein ZFP961 Represses Adipose Thermogenesis and Energy Expenditure through Interaction with PPARα

*Lei Huang, Pengpeng Liu, Qiyuan Yang, and Yong-Xu Wang**

Supplementary Information for

The KRAB Domain-containing Protein ZFP961 Represses Adipose Thermogenesis and Energy Expenditure through Interaction with PPARα

Lei Huang, Pengpeng Liu, Qiyuan Yang, and Yong-Xu Wang*

Department of Molecular, Cell and Cancer Biology, Program in Molecular Medicine, University of Massachusetts Medical School, 364 Plantation Street, Worcester, Massachusetts 01605, U.S.A.

*Correspondence: yongxu.wang@umassmed.edu

Phone: 508-856-5647

Fax: 508-856-4650

This file includes:

Supplementary Figures

Supplementary Figure 1

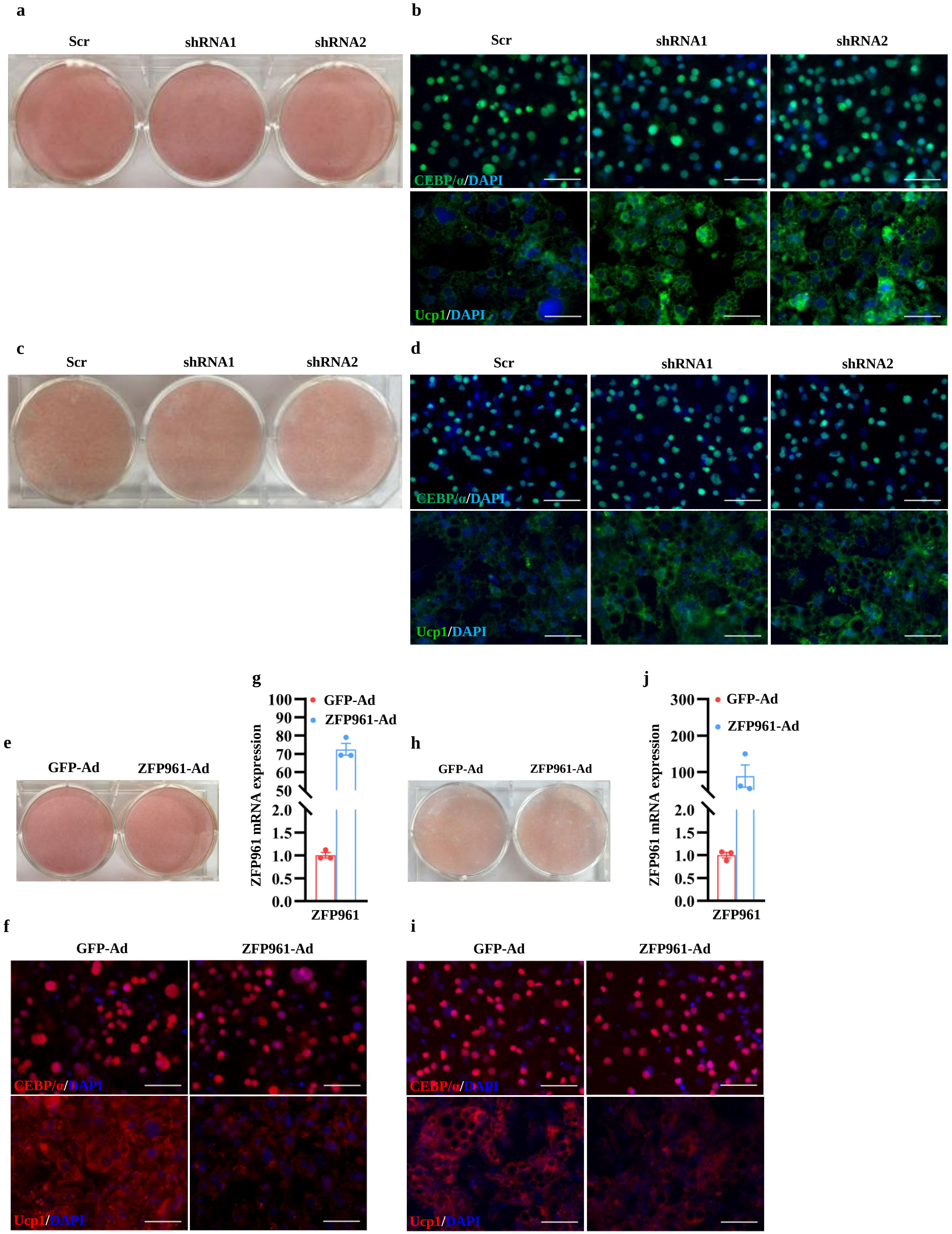

Figure S1. Immunostaining and adipocyte differentiation

a) Brown preadipocytes were infected with lentiviruses containing ZFP961 shRNAs and scramble (Scr) shRNA and differentiated. On day 6, the oil red O staining of triglycerides was performed. Representative pictures were shown. b) Brown adipocytes generated as in (a) and immunostaining of CEBP/α and Ucp1 were performed. Green=CEBP/α (upper panel) or Ucp1 (lower panel); blue=DAPI. Scale bar=200µm. c) Primary iWAT preadipocytes isolated from 2-weeks-old male mice were infected with lentiviruses containing ZFP961 shRNAs and Scr shRNA and differentiated. On day 6, the oil red O staining of triglycerides was performed. Representative pictures were shown. d) Primary iWAT adipocytes generated as in (c) and immunostaining of CEBP/α and Ucp1 were performed. Green=CEBP/α (upper panel) or Ucp1 (lower panel); blue=DAPI. Scale bar=200µm. e) Brown adipocytes were infected with adenovirus containing ZFP961 and GFP. On day 6, the oil red O staining of triglycerides was performed. Representative pictures were shown. f) Brown adipocytes generated as in (e) and immunostaining of CEBP/α and Ucp1 were performed. Red=CEBP/α (upper panel) or Ucp1 (lower panel); blue=DAPI. Scale bar=200µm. g) ZFP961 mRNA expression level was shown in mature brown adipocytes (n=3, per group). h) Primary iWAT preadipocytes were isolated from 2-weeks-old male mice and differentiated. The primary iWAT adipocytes were infected with adenovirus containing ZFP961 and GFP. On day 6, the oil red O staining of triglycerides was performed. Representative pictures were shown. i) Primary iWAT adipocytes generated as in (h) and immunostaining of CEBP/α and Ucp1 were performed. Red=CEBP/α (upper panel) or Ucp1 (lower panel); blue=DAPI. Scale bar=200 µm. j) ZFP961 mRNA expression level was shown in primary iWAT cells (n=3, per group).

Supplementary Figure 2

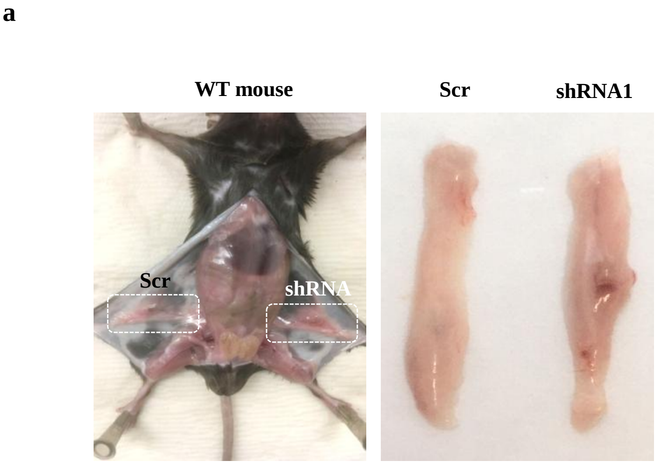

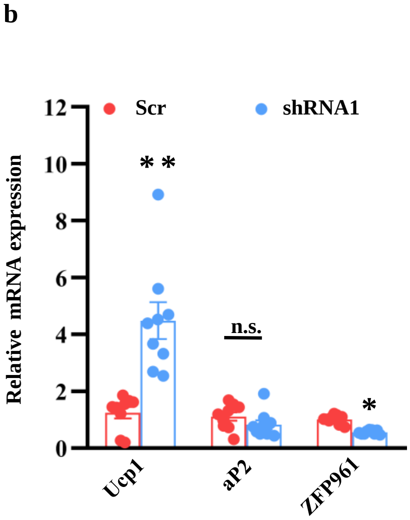

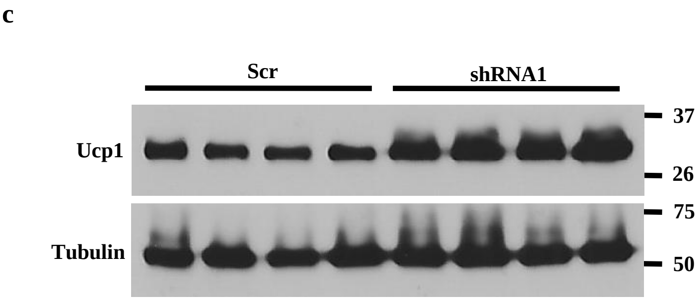

Figure S2. Acute silencing of ZFP961 remodels iWAT *in vivo*.

a) Representative image of wild-type male mice injected with lentivirus containing ZFP961-shRNA1 or Scr-shRNA to show gross iWAT appearance. b) Gene expression in iWAT of mice generated as in (a) (n=9, per group). c) Ucp1 protein in iWAT of mice generated as in (a) (n=4 per group). All error bars represent s.e.m. Two-tailed unpaired Student's *t*-test was performed. *$p < 0.05$; **$p < 0.01$; n.s, not significant

Supplementary Figure 3

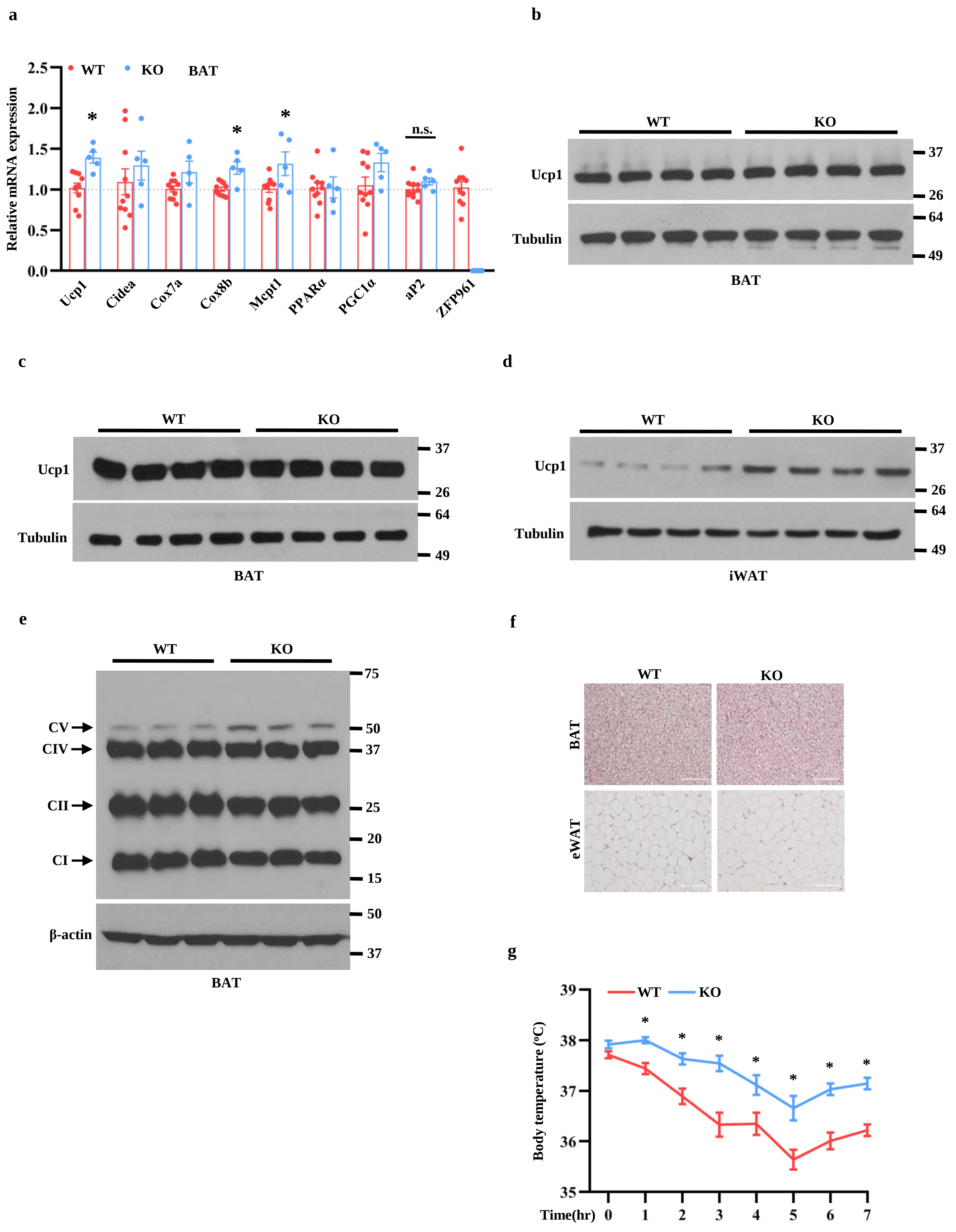

Figure S3. ZFP961 KO mice promotes adipose thermogenesis and energy expenditure

a) qRT-PCR analysis of gene expression in BAT of 10-week-old male KO mice (n=5, per group) and littermate control male mice (n=10, per group). b and c) Western blot analysis of Ucp1 protein expression in BAT of 10-week-old male (b) and female (c) KO mice and littermate control mice (n=4, per group). d) Western blot analysis of Ucp1 protein expression in iWAT of 10-week-old female KO mice and littermate control mice (n=4, per group). e) Western blot analysis of OXPHOS proteins expression in BAT of 10-week-old male KO mice and littermate control male mice (n=3, per group). f) H&E staining in BAT and eWAT of 12-week-old male KO mice and littermate control male mice. Representative images were shown (n=3, per group). Scale bar=200μm. g) Rectal core body temperatures of 16-week-old female KO mice (n=7, per group) and littermate control female mice (n=10, per group) upon cold stimulation at indicated time points. All error bars represent s.e.m. Two-tailed unpaired Student's *t*-test was performed. $*p < 0.05$; n.s, not significant

Supplementary Figure 4

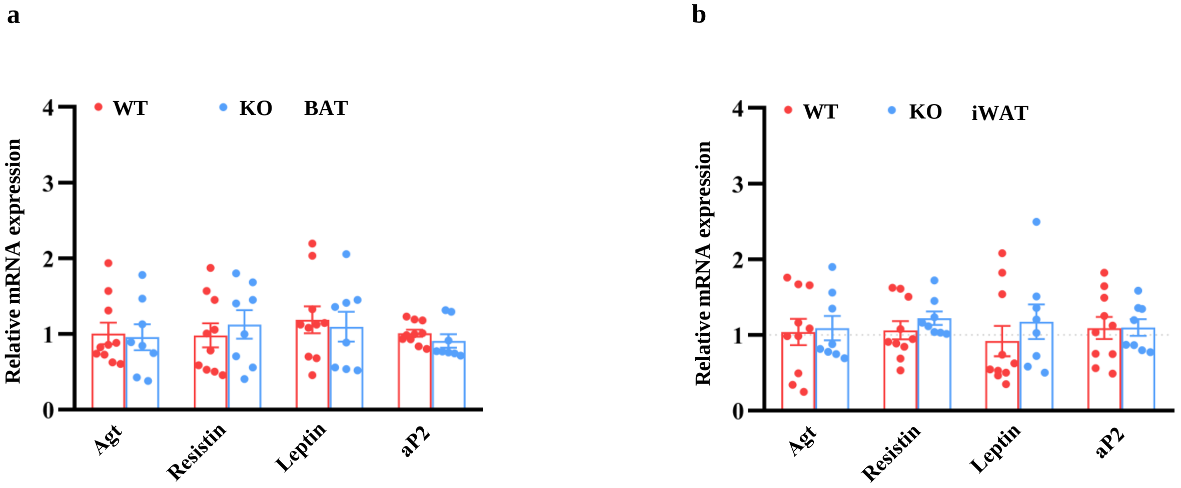

Figure S4. WAT-selective gene expression in BAT and iWAT

a and b) qRT-PCR analysis of WAT-selective gene expression in BAT (a) and iWAT (b) of 12-week-old male KO mice (n=8, per group) and littermate control male mice (n=10, per group).

Supplementary Figure 5

a

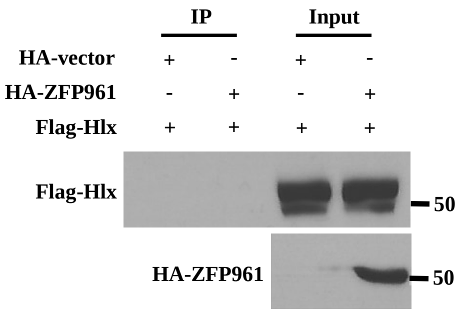

b

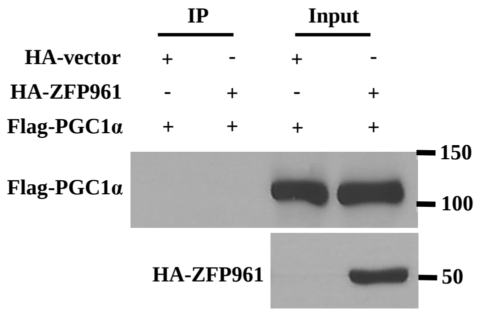

c

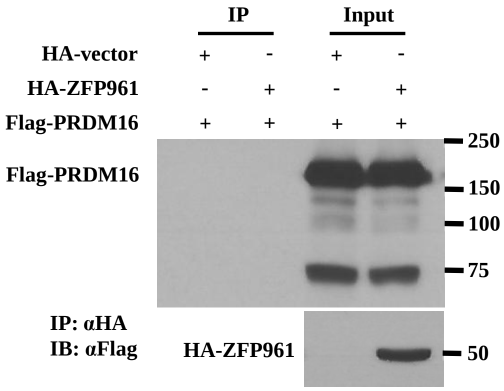

d

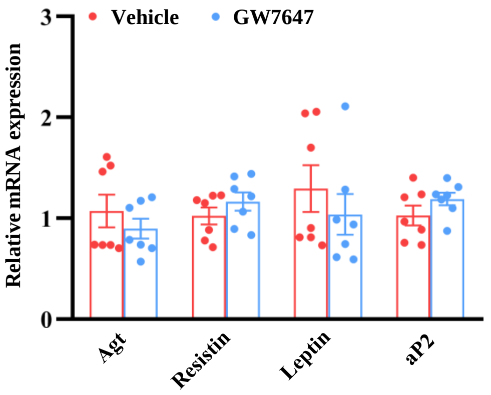

e

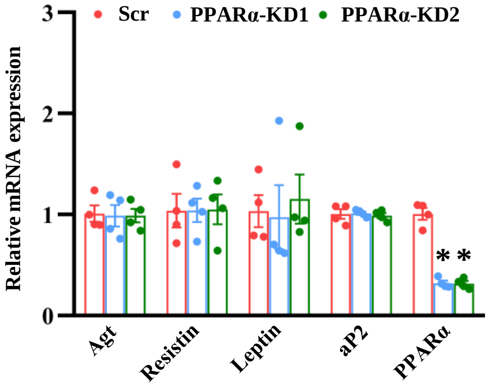

Figure S5. Co-immunoprecipitation analysis and WAT-selective genes expression

a-c) Co-immunoprecipitation analysis was carried out using cell extracts from HEK293T cells that were co-transfected with HA-ZFP961, Flag-Hlx (a), Flag-PGC1α (b), or Flag-PRDM16 (c). d) qRT-PCR analysis of WAT-selective genes expression in mature adipocytes treated with or without GW4767 (1 μM) (n=7, per group). e) qRT-PCR analysis of WAT-selective genes expression in PPARα-depletion mature adipocytes (n=4, per group). Two-tailed unpaired Student's *t*-test was performed. $*p < 0.05$

Supplementary Figure 6

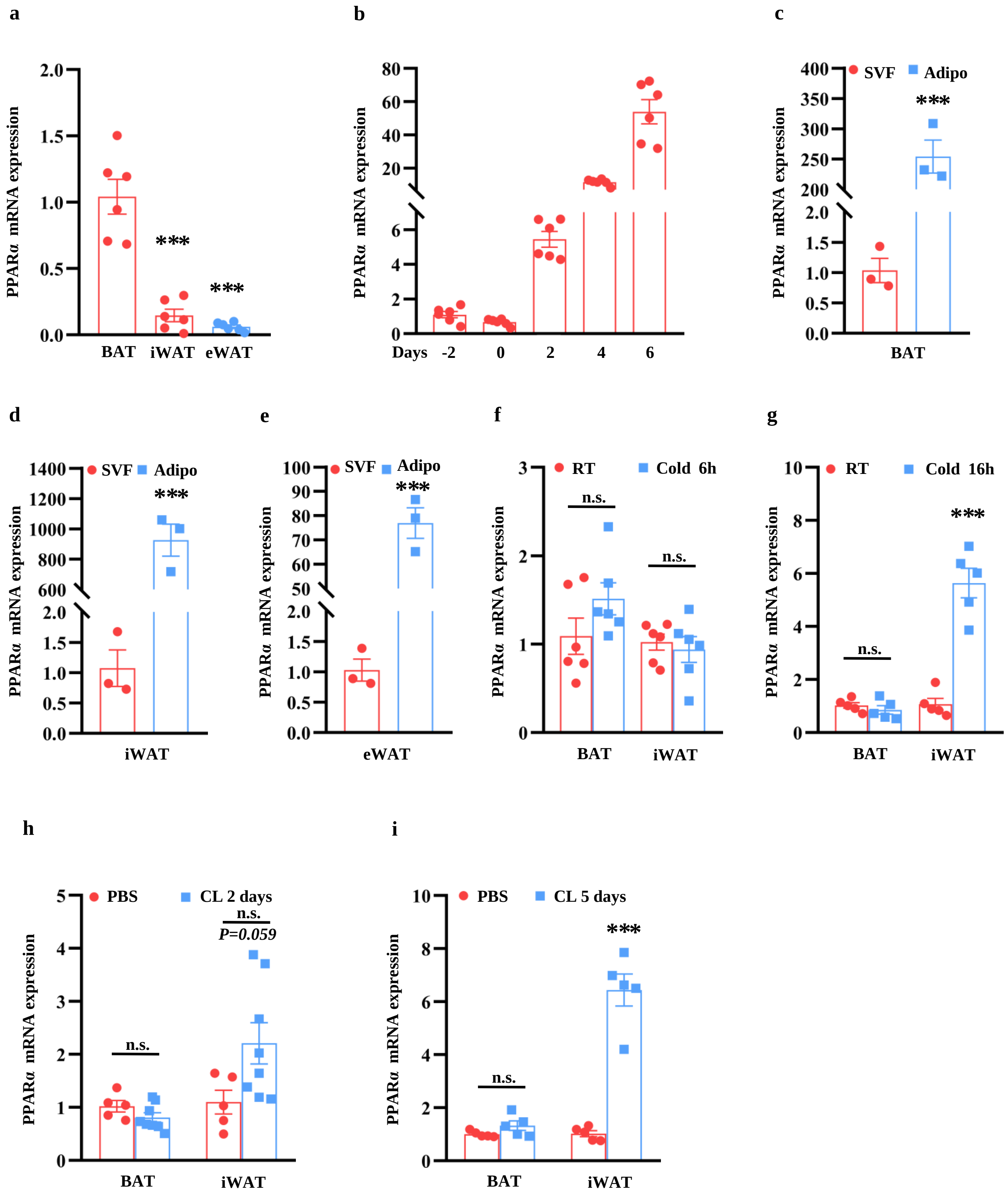

Figure S6. PPARα is abundant in BAT and induced by cold stimulation and β-adrenergic agonist in iWAT.

a) PPARα mRNA level analyzed by qRT-PCR in adipose tissue from 3-month-old male mice (n=6, per group). b) PPARα mRNA level during the differentiation of immortalized brown preadipocytes (n=6, per group). c-e) PPARα mRNA level in stromal vascular fraction (SVF) and mature adipocyte fraction of 3-month-old male mice (n=3, per group). f and g) PPARα mRNA level in BAT and iWAT of 3-month-old male mice at cold (4°C) for 6 h (f) (n=6, per group) and 16h (g) (n=5, per group). h and i) PPARα mRNA level in BAT and iWAT of 3-month-old male mice upon CL-316,243 treatment for two days (i) (n=5-8, per group) and five days (j) (n=5, per group). All error bars represent s.e.m. Two-tailed unpaired Student's *t*-test was performed. ***$p < 0.001$; n.s, not significant
